# Longitudinal Analysis of Binding Antibody Levels Against 39 Human Adenovirus Types in Sera from 60 Regular Blood Donors from Greifswald, Germany, over 5 Years from 2018 to 2022

**DOI:** 10.3390/v16111747

**Published:** 2024-11-07

**Authors:** Xiaoyan Wang, Konstanze Aurich, Wenli Zhang, Anja Ehrhardt, Andreas Greinacher, Wibke Bayer

**Affiliations:** 1 Institute for Virology, University Hospital Essen, University of Duisburg-Essen, 45147 Essen, Germany; xiaoyan.wang@uk-essen.de; 2 Institute for Transfusion Medicine, University Medicine Greifswald, 17475 Greifswald, Germany; konstanze.aurich@med.uni-greifswald.de (K.A.); andreas.greinacher@med.uni-greifswald.de (A.G.); 3 Virology and Microbiology, Center for Biomedical Education and Research (ZBAF), Faculty of Health, Witten/Herdecke University, 58455 Witten, Germany; wenli.zhang@uni-wh.de (W.Z.); anja.ehrhardt@uni-wh.de (A.E.)

**Keywords:** human adenovirus, HAdV, seroprevalence, binding antibodies, HAdV immunity

## Abstract

Adenoviruses are important human pathogens that are widespread and mainly associated with respiratory and gastrointestinal infections. In a previous study on human adenovirus (HAdV) seroprevalence, we observed reduced binding antibody levels against a range of HAdV types in sera collected from students in 2021 compared to sera collected before the SARS-CoV-2 pandemic. In this follow-up study, we wanted to verify this observation in a cohort of regular blood donors for whom serial samples were available. Therefore, HAdV-specific binding antibody levels were analyzed in sera collected over a 5-year period from 2018 to 2022 in a cohort of 60 regular donors to the blood bank of the University Hospital in Greifswald, Germany. Using ELISA-based assays, we quantified the binding antibody responses against 39 HAdV types. On the cohort level, we found largely stable antibody levels over the analyzed time period, with the highest antibody responses against HAdV-C1, -D25, -D26, -E4, -D10, -D27, -C5, -D75, -C2, and -C6. Only minor but significant reductions in comparison to the first serum samples from 2018 were detected for antibody levels in 2021 and 2022 against the low-prevalent types HAdV-A31, -D8, -D20, -D37, -D65, and -D69. On the other hand, we detected fluctuations in antibody levels on the individual level, with strong increases in antibody levels indicative of novel antigen contact. Interestingly, we frequently found simultaneous changes in antibody responses against multiple HAdV types, resulting in strong correlations of antibody responses against distinct clusters of HAdVs suggesting extensive cross-reactivity of HAdV-specific antibodies. To our knowledge, this is the first study of antibodies against a broad range of HAdV types in serum samples collected from a cohort of individuals over a prolonged period, and our data provide important insight into the long-term stability of HAdV-specific antibody levels. In this cohort of regular blood donors, we did not observe any major impact of the SARS-CoV-2 pandemic on HAdV immunity. Correlations of changes in antibody levels against different types indicate cross-reactivity of HAdV-specific antibodies that are important to consider for HAdV vector development. Our data also reveal possible candidates for future development of HAdV-based vectors.

## 1. Introduction

Human adenoviruses (HAdVs) are a large group of non-enveloped, double-stranded DNA viruses that are associated with respiratory, gastrointestinal, urogenital or ocular infections depending on the HAdV type (reviewed in [1]). HAdVs comprise more than 100 HAdV types as designated by the adenovirus working group based on genomic analyses [2] that are categorized into 7 species, A to G. While most HAdV infections are mild or can even be asymptomatic, some HAdV types are associated with severe disease; furthermore, HAdV infections by usually harmless types can become disseminated and life-threatening and are therefore considered a major threat in immunocompromised patients (reviewed in [1]).

On the other hand, HAdVs are an important tool for the development of vaccines, oncolytic vector treatments, and gene therapy. The successful use of HAdV-based vectors as vaccines against Ebola virus [3,4] and severe acute respiratory syndrome coronavirus 2 (SARS-CoV-2) [5,6,7] in recent years has demonstrated their high potential in this field.

An analysis of the seroprevalence of HAdVs is important both from an immunological point of view as well as for a potential use in vector development. Previously, studies on HAdV prevalence have mainly focused on one single type, or a small number of HAdV types (refer to [8] for a review of HAdV seroprevalence studies), although two large studies have been performed by Vogels et al. in a cohort of Belgian adults [9] and by D’Ambrosio et al. in a cohort of Italian children and young adults [10]. In our previous study, we therefore aimed to characterize the seroprevalence of a wide range of HAdV types, analyzing the binding and neutralizing antibody response against 39 HAdV types covering all 7 species, A to G, in serum samples from healthy medical student cohorts [11]. We found that both HAdV-specific binding and neutralizing antibody levels differed widely between the different HAdV types, with the highest binding antibody levels against HAdV-C1, -D33, -A31, -B35, -C5, -D26, -E4, and -B7, whereas the highest neutralizing antibody levels were detected against HAdV-C2, -B3, -C1, -F41, -G52, -C5, -A31, -E4, and -C6. When we analyzed two different student cohorts from October 2018 and April 2019, we found largely comparable levels of neutralizing antibodies; however, binding antibody levels in sera from a cohort from October 2021 were significantly lower for the highly prevalent HAdV types tested in this side-by-side comparison. However, since the analyses at the different time points were performed with sera from cohorts of different individuals, these results and their overall conclusions are limited.

To address the question how stable HAdV-specific antibody levels are over time, if significant declines in HAdV-specific binding antibodies could be observed during the SARS-CoV-2 pandemic when analyzing longitudinal samples from a consistent cohort and if there are HAdV type-specific differences, we performed the study presented here where consecutive serum samples obtained from regular blood bank donors were obtained covering the 5-year period from 2018 to 2022, with two serum samples obtained for each donor per year, which were tested for binding antibodies against 39 HAdV types. Our data show a general stability of the HAdV-specific antibody levels on the cohort level, with fluctuations of antibody levels on the individual level that show strong correlations of antibody levels against different clusters of HAdV types.

To our knowledge, this is the first report of a longitudinal study analyzing in detail the development of HAdV-specific antibody levels over time.

## 2. Materials and Methods

### 2.1. Viruses

In this study, we used a total of 39 different HAdV types covering species A to G, most of which were wild-type viruses, except for five types that were engineered as viral vectors encoding GFP and/or luciferase. For more details on how the HAdVs were obtained, cultured and purified, please refer to the previous publication [11].

### 2.2. Serum Samples and Ethics Approval

To analyze binding antibody levels against 39 HAdV types, longitudinal serum samples were obtained from a cohort of 60 blood donors selected from regular blood donors to the blood bank of the transfusion medicine of the University Hospital Greifswald. The donors were healthy adults fulfilling the requirements set forth by the European Directive 2002/98/EC [12] and implemented by the Guidelines for the Preparation of Blood and Blood Components and for the Use of Blood Products (German Hemotherapy Guidelines) [13]. Among other criteria, the blood donors were free of severe diseases including severe acute or chronic diseases of the immune, respiratory or gastrointestinal system, and free of infections with human immunodeficiency virus, hepatitis B virus, hepatitis C virus, and human T cell leukemia virus, and of previous or current other infections as specified in the guidelines. Furthermore, the blood donors had no current or recent infection in the four weeks prior to blood donation of unknown origin characterized by fever, diarrhea or other symptoms, or uncomplicated, mild flu-like infections within one week prior to blood donation. The blood donors had never received xenotransplants or animal cells and were not known or suspected users of injection drugs.

From 2018 to 2022, two serum samples per year were collected from each donor, with the first samples obtained between January and March and the second samples obtained between July and September. Only three samples were missing, specifically one each from donor 39 (N39-2019 sample 2), donor 43 (N43-2022 sample 2), and donor 44 (N44-2021 sample 1). In total, 597 serum samples were obtained. Serum samples were stored at −20 °C. Serum samples were anonymized, but information about the age and sex of the donors was preserved.

The use of the sera in this study received approval from the Ethics Committee of the Medical Faculty at the University of Greifswald (BB 014/14).

### 2.3. Binding Antibody ELISA

The binding antibody ELISA was conducted in 384-well Nunc MaxiSorp plates (Sigma-Aldrich, Taufkirchen, Germany) as described before [11]. Plates were coated with 2.5 × 10^7^ viral particles of purified, UV-inactivated HAdV in 25 μL phosphate-buffered saline (PBS) per well and incubated overnight at 4 °C. The plates were then washed with PBS containing 0.1% Tween 20 (PBS-T) and subsequently blocked with 75 μL/well of 20% fetal calf serum (FCS) in PBS for 5 h at room temperature. Following the blocking, 25 μL of serum samples diluted 1:1000 or 1:200 in PBS were added to the wells; the dilution was selected depending on the expected antibody level against the respective HAdV type. After overnight incubation at 4 °C, binding antibodies were detected with 25 μL/well of a horseradish peroxidase-labeled polyclonal donkey anti-human IgG antibody (Dianova, Hamburg, Germany), diluted 1:15,000 in PBS, and 25 μL/well of tetramethylbenzidine (TMB) substrate (1-step TMB Ultra; Thermo Fisher, Waltham, MA, USA). Each incubation step was followed by washing with PBS-T. The reaction was terminated by adding 25 μL/well of 1 N H_2_SO_4_, and the absorbance at 450 nm was measured using a Mithras2 microplate reader (Berthold Technologies, Bad Wildbad, Germany).

For the normalization of antibody levels in the samples, we adapted a method described before [14] and used serial two-fold dilutions of human IgG (Sigma-Aldrich, Taufkirchen, Germany) starting from 10 µg/mL as antibody standard that were included on every plate and treated in the same way as described above, but without coating with HAdV or the addition of human sera. Non-coated wells were used as background controls and treated in the same way as described above, including the addition of human sera. The background was calculated as the mean of at least 20 wells and used to determine the lower limit of detection for each HAdV type. The range of IgG standard concentrations that was used for calculation of the HAdV-specific antibodies was typically between 1 ng/mL and 78.125 ng/mL for all analyzed plates, translating to antibody reference concentrations between 1 µg/mL and 78.125 µg/mL for a 1:1000 dilution and 0.2 µg/mL and 15.625 µg/mL for a 1:200 dilution. Serum dilutions were adjusted for each individual HAdV type to ensure that the absorbance values of the samples were within the dynamic range of the respective ELISA assays. For detection of antibodies against HAdV types A18, A31, B14, B34, B50, D8, D17, D20, D37, D48, D65, D69, and F41, sera were diluted 1:200, for detection of antibodies against HAdV types A12, B3, B7, B11, B16, B21, B35, C1, C2, C5, C6, D9, D10, D13, D24, D25, D26, D27, D33, D70, D73, D74, D75, D80, E4, and G52, sera were diluted 1:1000.

### 2.4. Statistical Analysis

Data were visualized using GraphPad Prism software version 8.4.2 or R software version 4.3.2 and the ggplot package. One-way analysis of variance on ranks was performed using GraphPad Prism software version 8.4.2. Pearson correlation analysis was performed using R software version 4.3.2 and the stats package and the corrplot package for visualization.

## 3. Results

### 3.1. Characteristics of the Longitudinal Cohort

The cohort comprised 51 male (85.0%) and 9 female (15.0%) regular blood donors; the age of the male donors at the time of first sample collection ranged from 29 to 67 years (mean ± SD: 52.7 ± 10.2 years) and the age of the females from 32 to 66 years (mean ± SD: 46.9 ± 12.9 years).

### 3.2. Prevalence of 39 HAdV Types at the Beginning of the Study

**Figure 1:**
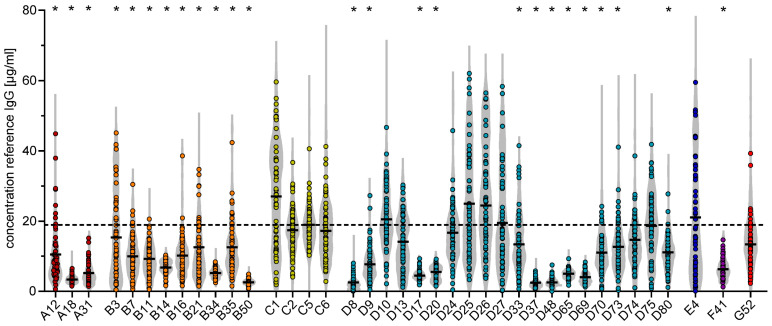
Binding antibody levels against 39 HAdV types in sera from 60 blood donors from 2018 to 2022. Sera of blood donors were analyzed for binding antibody levels against 39 HAdV types from species A to G, as indicated. Dots represent the values obtained for the first sera obtained in 2018, whereas the violin plots outline the distribution of the binding antibody values of all subsequent serum samples. Each dot indicates an individual serum sample. The black lines indicate the mean values of the first serum samples; the dashed line indicates the mean value of binding antibody levels against HAdV-C5. Statistically significant differences of the antibody levels in the first serum samples against the individual HAdV types compared to HAdV-C5 are indicated by * (*p* < 0.05; one-way analysis of variance on ranks with Dunn’s multiple comparison test).

Our data showed considerable variability in antibody levels across the 39 tested HAdV types with significant differences between antibody levels against the different HAdV types detected in the first samples collected at the beginning of 2018, which were largely preserved in the samples obtained thereafter (Figure 1, dot plot). In comparison to HAdV-C5, which is generally regarded as a highly prevalent HAdV type and can therefore serve as a reference type, the other species C HAdV types C1, C2, and C6 as well as the species D HAdV types D10, D13, D24, D25, D26, D27, D74, and D75, HAdV-E4, and HAdV-G52 showed binding antibody levels in a similar range. Notably, antibody levels against HAdV-C1, -D25, and -D26 tended to be higher than against HAdV-C5 but there was no statistical significance. On the other hand, the binding antibody levels against all tested species A and B HAdV types as well as the species D HAdV types D8, D9, D17, D20, D33, D37, D48, D65, D69, D70, D73, and D80 as well as HAdV-F41 were significantly lower in comparison to the binding antibody levels against HAdV-C5.

### 3.3. Dynamics of HAdV-Specific Antibody Levels over the Five-Year Observation Period

**Figure 2:**
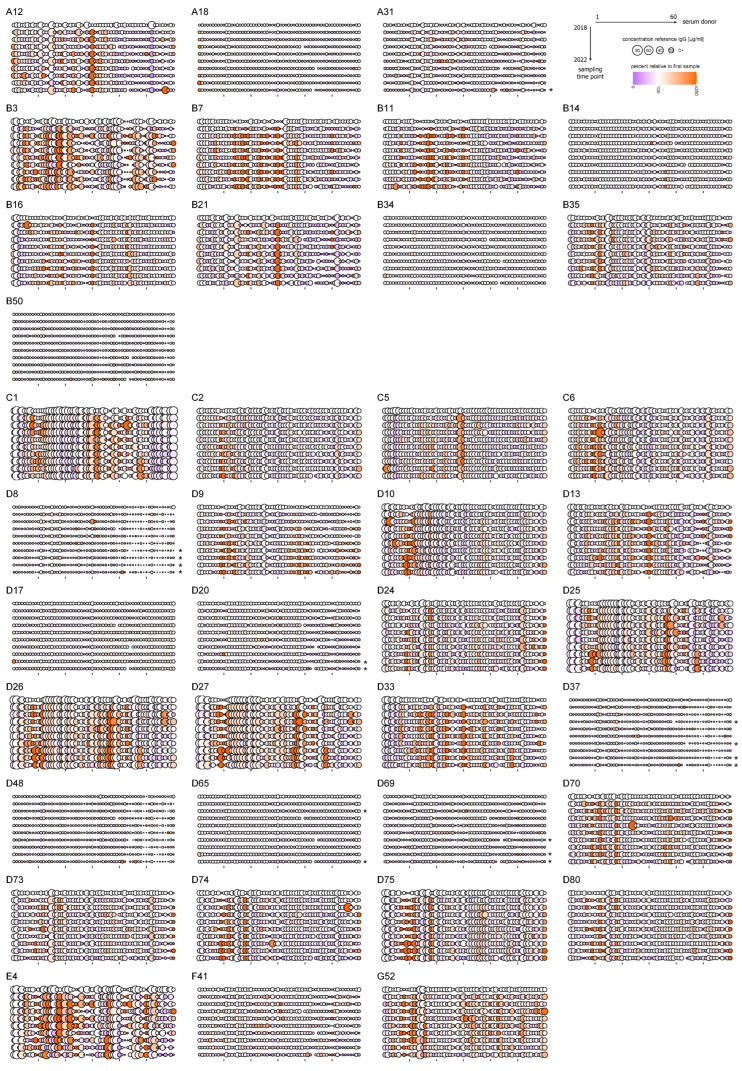
Bubble plot visualization of binding antibody levels over the observation period from 2018 to 2022. Binding antibody levels against the indicated HAdV types as shown in Figure 1. The binding antibody levels in reference to the IgG standard are indicated by the size of the bubbles; the color indicates the relative change in percentage compared to the respective level in the donor’s first sample obtained in early 2018. The bubble plots show donors ordered by their arbitrary donor number arranged from left to right and the collection dates from 2018 to 2022 arranged from top to bottom. Ticks on the x axis indicate the positions 10, 20, 30, 40, and 50. bAb: binding antibody. * Indicates statistically significant differences of the antibody levels in the serum samples from the indicated year compared to antibody levels in the first serum samples from 2018 (*p* < 0.05, one-way analysis of variance on ranks with Dunn’s multiple comparison test); no statistically significant differences were found for the comparison of the first set of samples from 2020 with samples from subsequent time points (*p* > 0.05).

In the samples obtained at the following time points, the overall binding antibody levels of the cohort were largely comparable to those observed for the first samples (Figure 1, underlaid violin plot). Only a few significant differences were observed for the low-prevalent types HAdV-A31, -D8, -D20, -D37, -D65, and -D69 in subsequent samples as shown in Table 1 and in the bubble plots shown in Figure 2 (see Supplementary Figure S1 for a heatmap representation): the antibody levels against HAdV-D37 and -D65 were significantly decreased at the second or first sampling time point in 2019, respectively, and the other significant decreases in antibody levels against HAdV-A31, -D8, -D20, -D37, -D65, and -D69 were found in the samples obtained at later time points in 2021 and 2022. The antibody levels against the other HAdV types did not show any significant differences, nor any noticeable trends towards reduced antibody levels over the studied time period. No statistically significant differences were found for the comparison of the samples collected right before the beginning of the SARS-CoV-2 pandemic, i.e., the samples collected in the first quarter of 2020, with samples from subsequent time points for any of the tested HAdV types.

However, changes in the antibody levels were observed on the individual level. For most HAdV types tested, we observed both rapid increases as well as mostly slow decreases in binding antibody levels in individual serum donors (Figure 2), which can be readily observed for those HAdV types where we found higher overall antibody levels such as HAdV types A12, B3, B7, B11, B21, C1, C5, C6, D10, D13, D24, D25, D26, D70, D74, D75, E4, and G52. These strong increases were suggestive of recent infection events. We also observed increases and fluctuations in the levels of antibodies against the lowest-prevalent HAdV types HAdV-A18, -B34, -B50, -D8, -D9, -D17, -D20, -D37, -D48, -D65, and -D69, but here antibody levels remained at very low levels. On the other hand, and regardless of the antibody level, many donors showed remarkably stable antibody levels over the five-year observation period, which can be readily seen for many donors’ antibody levels against some highly prevalent HAdV types such as C1, C2, and C5 but also for lower-prevalence types such as B14 and B34.

It is important to note that increases in antibody levels were not only observed for those donors who had low antibody levels against particular HAdV types at the start of the study, but also for those with intermediate to high levels, as is most prominently seen for HAdV-B3, -C1, -C5, -D26, -D73, and -E4 (refer to Supplementary Figure S2 for a bubble plot visualization with the serum donors ordered by descending antibody levels for each HAdV type). For many sera with high binding antibody levels at the beginning of the study, we observed a slow decline of antibody levels in the following samples, suggesting a possible recent infection event before the study period. In most cases, however, high antibody levels after a putative infection event during the study period were stable for a long time, with a few notable exceptions observed; for example, in the antibody levels against HAdV-A12 (donors 5, 12, 18, 25, 26, 33, 50, 56, 60), -A31 (donors 19, 21, 24, 29, 32, 46, 58), -B3 (donors 17, 24, 30, 43, 58, 60), -B14 (donors 31, 58), -B16 (donors 6, 7, 24), -B35 (donors 5, 17), -C1 (donor 43), -C2 (donor 18), -C5 (donor 2), -C6 (donor 12), -D9 (donor 18), -D10 (donors 3, 5, 18), -D70 (donors 24, 38), -D73 (donor 12), -D74 (donors 28, 56), and -E4 (donors 27, 30, 44, 54, 60) (Figure 2), where we observed very short peaks in antibody levels in only one respective sample.

### 3.4. Correlation Between Changes in HAdV-Specific Antibody Levels

**Figure 3:**
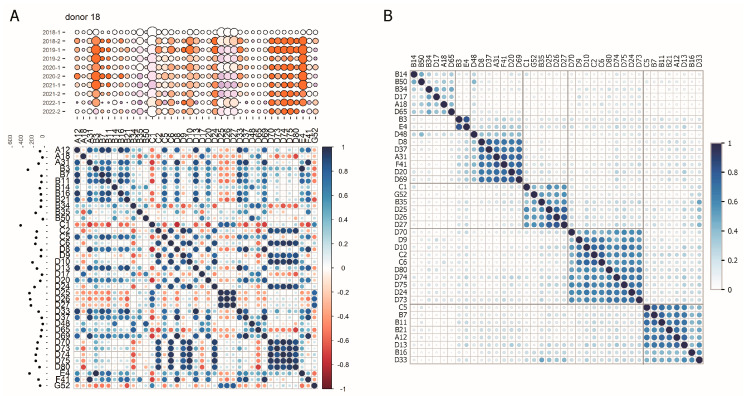
Correlation analysis of binding antibody levels. (**A**) A Pearson correlation analysis was performed for the binding antibody levels against the different HAdV types, as shown in Figure 2 for each individual donor. Shown are the correlation maps for the indicated selected donors (for all donors, please refer to Supplementary Figure S2) with the binding antibody levels against the indicated HAdV types shown as bubble plots as in Figure 2 (binding antibody levels in reference to the IgG standard are indicated by the size of the bubbles; color indicates the relative change in percentage compared to the respective level in the donor’s first sample obtained in early 2018), on top of the correlation map, and the area under the curve of the binding antibody levels over the 5-year period shown on the left side of the correlation map. (**B**) A mean correlation matrix for the cohort was calculated from the individual correlation matrices per donor. Shown is a clustered correlation heatmap created using the average agglomeration clustering method.

It is noteworthy that when the changes of antibody levels over time were analyzed, very similar patterns were observed for antibodies against multiple HAdV types, such as HAdV-D25, -D26, and -D27, or HAdV-D24, -D73, -D75, and -D80 (Figure 2). We therefore performed a correlation analysis of the antibody levels observed in the individual donors for the different HAdV types (Figure 3A and Supplementary Figure S3). As seen in the representative plot for donor 18 (Figure 3A), high levels of correlation are due to simultaneous changes in antibody levels, rather than the antibody levels as such, since we found a strong correlation of the antibody responses against HAdV types D25, D26, and D27, but a much weaker correlation of these three species D types with HAdV C1, which also started at a high level in the first sample but showed different changes over time. Furthermore, we also observed high correlation values for antibody responses that were present at lower levels, such as those against HAdV-A12, -A31, -B7, -B11, -B16, and -B21. Similar observations were made for the other donors (Supplementary Figure S3): increases or decreases in antibody levels were not limited to one individual HAdV type but were observed simultaneously for multiple HAdV types, resulting in strongly positive correlations. These strongly positive correlations were particularly pronounced and most consistent for antibody responses against HAdV-D25, -D26, and -D27 (most pronounced in donors 8, 9, 12, 33, 36, 37, 38, 40, 43, 48, 57), and included a positive correlation with antibody responses against other types such as HAdV-C1, HAdV-E4 or HAdV-G52 in some but not all serum donors. Furthermore, the analysis revealed a strong correlation of antibody levels against HAdV-A12, -B7, -B11, -B16, -B21, -D13, and -D33 (most pronounced in donors 1, 12, 18, 19, 23, 25, 30, 32, 37), of antibody levels against HAdV-C2, -C6, -D9, -D10, -D24, -D70, -D73, -D74, -D75, and -D80 (most pronounced in donors 8, 9, 10, 13, 14, 15, 18, 60), and of antibody levels against HAdV-B3 and -E4 (most pronounced in donors 6, 7, 9, 11, 13, 14, 15, 17, 18, 19, 22, 30, 33, 44, 46, 48, 50, 60). Another correlation was observed between antibody levels against HAdV-A31, -D8, -D20, -D37, -D48, -D69, and -F41 (most pronounced in donors 5, 14, 21, 22, 24, 26, 41, 42, 50, 53, 56), in spite of comparably low antibody levels against these HAdV types. While it cannot be ruled out that multiple HAdV infections occurred in the time between two sample collections, these parallel changes in antibody levels, especially those observed in most donors, are highly indicative of cross-reactivity between antibodies induced by HAdV.

The correlations observed for the individual donors were also observed when the average of the cohort was calculated, as is shown in Figure 3B and Table 2, with the abovementioned clustering of HAdV types: HAdV-B3 and -E4 are in one cluster with relatively weak correlations to any of the other HAdV types, with the other larger clusters consisting of HAdV-D8, -D37, -A31, -F41, -D20, and -D69, of HAdV-C1, -G52, -B35, -D25, -D26, and -D27, of HAdV-D70, -D9, -D10, -C2, -C6, -D80, -D74, -D75, -D24, and -D73, and of HAdV-C5, -B7, -B11, -B21, -A12, -D13, -B16, and -D33.

## 4. Discussion

Our data obtained in this study provide detailed insight into the course of HAdV-specific antibody levels over a five-year period, demonstrating a largely stable maintenance on the cohort level and fluctuations on the individual level. Strong increases in antibody levels on the individual level are suggestive of HAdV infection events, and parallel increases in antibody levels against multiple HAdV types after putative infection events suggest cross-reactivity of HAdV-specific antibodies.

Once again, our data show that while all donors had readily detectable antibodies against HAdV-C5, which is generally regarded as highly prevalent [15], the antibody levels against the other species C HAdV and against a number of species, B, D, E, and G viruses, were equally high, including HAdV-D26, which is regarded as a low-prevalence type [16]. These results are in accordance with our previous data from the studies of HAdV-specific antibody responses in sera from healthy student cohorts [11] or from patients with neuromuscular diseases [17]. In those previous studies, we also observed rather high binding antibody levels against HAdV-D26 but low neutralizing antibody levels, which have led in the past to the characterization of HAdV-D26 as a rare HAdV type [16]. Interestingly, there are some differences between the three cohorts: the binding antibody level against HAdV-A31 was rather low in the blood donor and the NMD patient cohort, whereas it was remarkably high in the student cohort. Similarly, the HAdV-B50-specific antibody levels in the blood donor cohort and the student cohort were very low, whereas they were quite high in the NMD patient cohort. The binding antibody response against HAdV-C6 was comparably high in the blood donor and NMD patient cohorts, whereas we found rather low levels in the student cohort. These different patterns in the different cohorts indicate that multiple factors are influencing the prevalence of HAdV-specific antibodies. Other studies have mainly focused on the prevalence of neutralizing antibodies, and therefore a direct comparison with our results is not possible. In a large study of neutralizing antibodies against 47 HAdV types in healthy adults, it was shown that the highest frequencies of positive sera were observed against the HAdV types C1, A31, A12, B3, and C2, and the lowest frequencies were observed against most species B and D types [9]. This does not reflect our findings for the binding antibody response since we observed only low levels of antibodies against species A, but high levels against some of the tested species D HAdV types. Another large-scale study analyzed the prevalence of neutralizing antibodies against 33 HAdV types in children, and found the highest frequency of neutralizing sera against the HAdV types C2, C5, C1, B3, C6, A31, A18, E4, and B7 [10], which again does not reflect our findings with regard to the species A HAdV types.

With regard to the longevity of HAdV-specific immune responses, some data from vaccine or gene therapy trials in humans are available. Early studies of inactivated HAdV-B3, -B7, and -E4 vaccines demonstrated largely stable antibody levels from 3 weeks after vaccination until the end of observation 5 months after vaccination [18,19]. In a study of a live enteric HAdV-B21 vaccine, neutralizing antibody responses were shown to decrease to about 50% of the level four weeks after vaccination by four months after vaccination [20]. Data from a gene therapy trial using HAdV-C5 showed stable vector-boosted anti-HAdV-C5 binding and neutralizing antibody responses for approximately three months of observation after therapy [21]. Recently, data from a phase III trial of an HAdV-C5-based vaccine against severe acute respiratory syndrome coronavirus 2 (SARS-CoV-2) demonstrated a steady HAdV-C5-neutralizing antibody response in vaccine and placebo recipients for a 6-month observation period (vaccine recipients reached an elevated level 1 month after vaccination that was maintained [22]). These data indicate that HAdV-specific antibody responses are generally stable over a longer time period, similar to what we observed in our study on the cohort level. The data covering the longest time frame comes from a study of the immune response to the United States Food and Drug Administration-licensed live HAdV-E4, -B7 vaccine, where it was shown on the cohort level that the neutralizing antibody response against both viruses was stable for as long as six years after immunization [23]. These data seem at first to be in contrast to the pronounced fluctuations that we observed in the binding antibody levels, particularly for HAdV-E4, but it has to be pointed out that on the cohort level, we also did not detect significant changes in the HAdV-E4 binding antibody levels, because of individual fluctuations balancing out each other.

Interestingly, we did not observe a striking effect of the SARS-CoV-2 pandemic on the binding antibody levels against HAdV in this longitudinal study, as we had observed before in the student cohort [11]. While we did observe reduced antibody levels in some low-prevalence types in the 2021–2022 samples compared to the first samples from 2018, most donors showed stable antibody levels that were not markedly reduced during the SARS-CoV-2 pandemic against most HAdV types that we tested. This discrepancy might be due to the different ages of the serum donors: the mean age of the student cohorts that we analyzed in our previous study was 23.5 years (October 2018 cohort) and 23.0 years (October 2021 cohort; [11]) compared to a mean age of 51.8 years of the blood donor cohort analyzed in the present study. While we did not observe a direct significant impact of age on the antibody levels against HAdV in our previous study in a cohort of patients with neuromuscular diseases [17], the much higher age of the donors in this longitudinal study may have impacted not the level but the steadiness of the HAdV-specific antibody responses. It has been shown before that the seroprevalence of HAdV is higher in adults compared to children for some but not all HAdV types, probably due to continued or repeated exposure to some HAdV types [10,24], which may have contributed to increased steadiness of HAdV-specific antibody levels. On the other hand, it has been shown that the incidence of HAdV infections was less reduced than that of other respiratory viral infections such as influenza virus, parainfluenza virus or respiratory syncytial virus infections during the SARS-CoV-2 pandemic [25,26,27,28], which may also have contributed to the maintenance of the HAdV-specific antibody levels.

With regard to the long-term stability of binding antibody levels, it may also be mentioned that it cannot be determined if a long-term stable antibody level is due to an inherent stability *per se* or due to continuous or repeated restimulation. A factor that has to be considered as maybe underlying the long-term stability of binding antibody levels that we observed in this longitudinal study is the possibility of persistent infections that HAdVs have been described to establish. For instance, it has been shown, mainly in the context of severe disease in immunocompromised children, that HAdVs can establish a latent infection of gut-associated lymphocytes and reactivate from these cells under immunosuppression, which leads to high-level replication in gut epithelia [29,30]. Interestingly, gut-associated persistence of HAdV in adults has not been described, but the frequent isolation of HAdV from tonsillar tissues suggest that these may be another site of HAdV persistence [30], possibly also in adults. In a study of the persistence of species C HAdVs in human tonsil and adenoid tissues, Garnett et al. described a decline in HAdV DNA depending on the age in children and young adults, and suggested that these latent HAdV infections are cleared when reaching the age of young adults [31]. The degree to which persistent or latent HAdV infections are established, and whether all HAdV types establish persistent infections and at similar rates and in similar locations, is currently unknown. Our data show that relatively high antibody levels are stable over a prolonged time, which could be indicative of a continuous stimulation by occasionally reactivating or low-level replicating virus.

The side-by-side analysis of the antibody responses against this large number of HAdV types over a longer period of time also clearly revealed strong correlations of antibody responses against different clusters of HAdV types, which are highly indicative of cross-reactivity of the HAdV-specific antibody responses. It should be noted in this context that the classification of HAdV types by the International Committee on the Taxonomy of Viruses, which has so far only recognized HAdV types 1 through 54 as being distinct types, includes a low level of cross-reactivity of neutralizing antibodies against other HAdV types as a criterion for the assignation of a distinct HAdV type, but not HAdV-specific binding antibodies [32]. Our data indicate that the cross-reactivity of binding antibodies is broader and not limited to reactivity within the HAdV species. It should therefore also be pointed out that the dynamics of binding antibodies observed in the sera cannot be interpreted as indicative of an infection with a particular HAdV type. We observed correlations in antibody reactivities for HAdV types irrespective of the antibody level, but depending on simultaneous changes in antibody levels. Our results of cross-reactivity of binding antibodies is partly in agreement with a previous report on increases in neutralizing antibodies against HAdV types 1 through 10 in acutely infected individuals, that also described simultaneous increases in neutralizing antibody levels against HAdV-B3 and -E4, and of antibody levels against HAdV-C5 and -B7 [33]. They also described further simultaneous increases that we did not observe in our study, which is probably due to differences in the cross-reactivities of binding and neutralizing antibodies. It has previously been shown that HAdV-binding antibodies are specific for the major capsid protein hexon, penton, and fiber [34,35,36,37,38] but also minor capsid proteins pIIIa [39] and pIX [40], and all these different reactivities may contribute to varying degrees to both the reactivity and probable cross-reactivity observed in our study. Some of the assumed cross-reactivities can be readily explained by the close relatedness of the viruses such as HAdV-D25, -D26, and -D27, whereas others do not show a high degree of sequence similarity, such as HAdV-B3 and HAdV-E4. A conclusive analysis of cross-reactivities would require confirmed knowledge of individual HAdV infection events, and would most easily and convincingly be achieved in an animal model, where single HAdV types can be administered to previously non-exposed animals and resulting anti-HAdV antibodies tested for cross-reactivities, which we aim to perform in the future.

There are a few limitations to our study: the current cohort was not sex-balanced and comprised only a few women; however, we have previously not observed any differences between males and females in antibody levels against HAdV [11], which lets us conclude that the skewed distribution of donor sex did not impact the study results. Furthermore, there is no information available on a possible immunization of the blood donors against SARS-CoV-2 with an HAdV-based vector in 2021; an increase in HAdV-D26 specific antibody levels in the second samples of 2021 in a few donors (donors 9, 36, 48) might be an indication of an immunization with the HAdV-D26-based Janssen vaccine Jcovden [6]. It would be of great interest to analyze serial samples from HAdV-based vaccine recipients in the future to obtain more conclusive results on the cross-reactivity of HAdV-based vaccine-induced antibodies. Finally, young age groups are not represented by this study, and studying the development of HAdV-specific antibodies in children over a prolonged period of time may be especially interesting.

## 5. Conclusions

This study provides detailed insight into the long-term development and stability of HAdV-specific binding antibody levels, covering a five-year period and reactivity against 39 HAdV types, which does not show a strong effect of the SARS-CoV-2 pandemic on HAdV-specific immune levels and provides insight into HAdV immunology and possible use of individual HAdV types for future therapeutics development.

## Figures and Tables

**Table 1:** Overview of binding antibody levels over the observation period from 2018 to 2022. Binding antibody levels in sera were determined by ELISA, and the mean binding antibody levels relative to the reference IgG in µg/mL, and standard deviations were calculated. Bold type indicates statistically significant differences of the antibody levels in the serum samples from the indicated collection time point compared to antibody levels in the first serum samples from 2018 (*p* < 0.05, one-way analysis of variance on ranks with Dunn’s multiple comparison test); no statistically significant differences were found for the comparison of the first samples from 2020 with samples from subsequent time points (*p* > 0.05). 1st: first sample of that year; 2nd: second sample of that year.

	2018				2019				2020				2021				2022			
	1st		2nd		1st		2nd		1st		2nd		1st		2nd		1st		2nd	
	Mean	SD	Mean	SD	Mean	SD	Mean	SD	Mean	SD	Mean	SD	Mean	SD	Mean	SD	Mean	SD	Mean	SD
A12	10.6	8.5	9.2	8.5	9.9	7.6	9.1	7.9	10.0	7.9	10.1	8.9	9.5	7.4	9.9	8.1	8.9	6.6	9.6	8.8
A18	3.4	1.0	2.9	1.0	3.0	1.1	3.1	1.1	3.0	1.0	3.1	1.1	3.1	0.9	3.2	1.0	3.5	1.4	3.0	0.9
A31	5.2	3.2	4.7	3.1	5.0	3.2	4.6	2.9	4.8	2.9	4.5	2.8	4.4	3.0	4.4	2.9	3.8	2.6	**3.8**	**3.0**
B3	15.4	11.7	12.9	10.7	18.5	13.8	15.6	12.9	16.8	13.4	17.6	13.6	15.1	12.6	16.8	13.7	16.3	12.5	16.4	13.2
B7	10.0	6.0	8.6	5.5	10.3	4.7	10.0	5.6	10.6	5.5	10.9	6.2	10.2	6.0	11.3	5.7	10.4	6.0	9.9	5.7
B11	9.3	4.7	7.5	4.2	8.3	3.5	7.7	4.0	8.4	4.3	9.0	4.4	8.8	4.6	8.7	4.0	8.7	4.7	9.4	5.3
B14	6.8	2.0	6.5	2.2	6.8	2.2	6.6	2.1	6.5	2.2	6.5	2.1	6.2	2.1	6.6	2.0	6.5	2.0	6.6	1.9
B16	10.2	5.9	9.6	7.1	10.2	5.2	9.2	5.4	9.4	5.6	10.0	6.2	9.6	5.7	10.5	6.0	9.9	5.4	10.0	5.7
B21	12.6	7.9	10.8	8.1	11.8	7.8	10.4	7.9	11.0	7.8	11.2	9.1	10.5	8.7	10.4	7.4	9.7	6.8	10.2	8.2
B34	5.2	1.3	4.7	1.4	4.8	1.4	5.0	1.5	5.0	1.5	5.1	1.4	4.9	1.3	4.8	1.3	4.9	1.6	4.6	1.3
B35	12.6	7.4	12.1	7.5	12.7	7.4	13.6	8.1	12.6	8.2	12.6	7.9	12.0	7.9	12.7	7.3	11.3	6.6	10.5	6.1
B50	2.6	0.8	2.2	0.7	2.3	0.8	2.3	0.8	2.2	0.8	2.2	0.8	2.2	0.8	2.4	0.8	2.3	0.9	2.5	1.1
C1	27.0	15.8	27.9	16.1	30.5	15.5	27.7	14.6	26.6	15.0	26.5	14.9	26.9	13.6	27.2	13.8	26.4	14.2	26.0	14.6
C2	17.4	6.5	16.2	6.4	16.6	5.7	16.8	6.8	16.5	6.5	17.5	6.3	16.5	6.6	16.3	6.8	17.2	6.7	16.3	6.9
C5	18.9	5.4	18.2	7.2	18.5	6.3	17.7	6.5	18.5	6.4	19.2	6.5	18.3	6.2	19.1	6.7	19.0	7.8	18.2	5.9
C6	17.3	8.8	16.6	9.1	17.2	8.4	18.1	11.2	17.4	8.5	18.8	9.0	17.3	8.5	17.4	9.8	18.5	9.0	17.4	9.2
D8	2.5	1.7	2.1	1.6	2.3	2.4	1.9	1.5	2.1	1.7	1.9	1.5	**1.8**	**1.6**	**1.7**	**1.6**	**1.5**	**1.4**	**1.7**	**1.6**
D9	7.7	5.3	6.5	4.7	6.8	4.4	7.4	4.8	7.5	4.7	8.6	5.0	8.4	4.7	8.2	5.1	8.9	5.3	8.4	4.6
D10	20.6	9.3	19.4	9.1	21.2	10.3	19.9	8.7	20.7	8.3	21.9	9.4	19.6	7.1	19.9	8.5	20.6	8.6	18.9	8.3
D13	14.2	7.7	12.4	7.4	14.4	7.4	13.1	8.3	14.0	7.9	13.5	8.2	12.7	7.7	14.0	8.1	12.6	7.2	12.6	7.8
D17	4.5	1.5	3.9	1.2	4.0	1.1	4.4	1.2	4.5	1.3	4.7	1.3	4.5	1.2	4.5	1.2	4.5	1.4	4.1	1.2
D20	5.5	1.8	5.0	2.1	5.1	2.0	4.7	1.9	5.0	2.2	4.8	2.3	4.5	2.0	4.7	2.1	**4.3**	**2.0**	**4.3**	**2.0**
D24	16.7	7.9	15.5	9.3	16.5	8.4	15.6	8.7	16.3	7.1	17.4	8.7	16.8	7.8	16.5	8.5	16.8	8.1	15.4	8.4
D25	25.0	14.9	25.1	15.8	25.8	15.0	25.2	15.6	23.9	15.8	23.5	15.0	24.4	14.4	26.5	15.2	24.8	14.8	24.2	15.4
D26	24.5	14.1	25.9	15.1	28.4	15.1	28.7	15.4	27.7	15.4	26.7	14.7	27.7	14.1	29.5	14.9	28.3	15.1	27.0	15.2
D27	19.5	13.9	20.8	14.6	22.3	14.2	22.5	14.8	21.2	15.0	20.5	14.4	20.9	14.6	23.2	14.9	21.0	14.4	20.1	14.6
D33	13.4	8.7	13.0	8.5	14.8	7.8	14.0	7.6	14.3	8.6	14.2	7.8	12.9	7.3	14.3	7.7	13.2	7.4	12.8	6.9
D37	2.5	1.3	2.1	1.3	2.2	1.5	**1.8**	**1.3**	2.1	1.4	1.8	1.2	**1.7**	**1.2**	1.9	1.3	**1.7**	**1.2**	**1.8**	**1.3**
D48	2.5	1.3	1.9	1.2	2.1	1.1	2.1	1.1	2.1	1.1	2.0	1.0	1.9	1.1	1.9	0.9	2.0	1.1	2.0	1.2
D65	5.1	1.3	4.6	1.3	**4.4**	**1.2**	4.7	1.4	4.6	1.2	4.7	1.3	4.5	1.2	4.6	1.2	4.5	1.5	**4.3**	**1.1**
D69	4.0	1.6	3.5	1.6	3.6	1.6	3.3	1.4	3.5	1.6	3.3	1.5	**3.0**	**1.4**	3.2	1.5	**3.0**	**1.3**	**2.8**	**1.5**
D70	11.1	5.4	11.2	6.2	13.4	6.3	11.9	5.7	10.4	7.3	10.9	5.2	10.9	5.1	11.5	5.6	10.8	5.4	9.7	5.1
D73	12.7	7.1	11.9	9.0	12.9	7.7	12.5	8.1	12.4	6.8	13.4	8.5	11.9	7.1	11.5	7.8	12.1	7.1	10.4	7.8
D74	14.7	7.0	13.7	7.7	15.0	8.8	13.0	7.0	13.7	6.5	14.3	7.3	13.1	6.6	14.1	7.6	13.6	7.2	12.1	6.9
D75	18.7	9.8	17.6	10.2	19.3	9.2	18.0	10.0	18.4	8.8	19.0	9.5	17.7	8.7	19.3	9.2	19.9	9.3	17.7	9.6
D80	11.1	5.0	10.7	5.7	10.8	5.0	10.3	5.5	11.0	5.0	12.4	5.6	11.0	5.0	10.8	4.8	10.8	5.1	9.6	5.7
E4	21.1	16.1	19.3	15.5	24.2	17.3	20.6	15.5	22.8	16.9	21.9	16.0	18.2	15.5	19.2	17.0	18.0	16.2	16.5	16.5
F41	6.3	2.9	5.8	3.1	6.3	3.1	6.1	2.9	6.6	3.0	6.3	2.8	5.9	2.8	5.9	2.8	5.3	2.6	5.1	3.0
G52	13.4	6.8	13.9	7.7	14.7	7.5	16.0	8.7	15.4	9.1	15.7	8.0	15.6	7.6	15.4	8.9	13.4	7.1	12.4	7.9

**Table 2:** Correlation analysis of binding antibody levels on the cohort level. Shown are those HAdV types where we found a moderate correlation (correlation coefficient 0.3–0.49), a strong correlation (correlation coefficient 0.5–0.79) or a very strong correlation (correlation coefficient ≥ 0.8) of the binding antibody levels.

	Moderate Correlation	Strong Correlation	Very Strong Correlation
A12	B16, D33	B7, B11, B21, C5, D13	
A18	B34, B50, D17, D65		
A31	D48, E4	D8, D20, D37, D69	F41
B3	F41		E4
B7	B16, D33	A12, B11, B21, C5, D13	
B11	B16, D33	A12, B7, B21, C5, D13	
B14	B34, B50, D65		
B16	A12, B7, B11, B21, C5, D13	D33	
B21	B16, D33	A12, B7, B11, C5, D13	
B34	A18, B14, D17, D65		
B35	C1, D33	D25, D26, D27, G52	
B50	A18, B14, D48		
C1	B35, G52	D25, D26, D27	
C2	D70	C6, D9, D10, D24, D73, D74, D75, D80	
C5	B16, D33	A12, B7, B11, B21, D13	
C6	D70	C2, D9, D10, D24, D73, D74, D75, D80	
D8	D48	A31, D20, D37, D69, F41	
D9	D70, D74	C2, C6, D10, D24, D73, D75, D80	
D10	D13, D33	C2, C6, D9, D24, D70, D73, D74, D75, D80	
D13	B16, C2, D10, D20, D74, D75	A12, B7, B11, B21, C5, D33	
D17	A18, B34, D65		
D20	D13, D48, E4	A31, D8, D37, D69, F41	
D24		C2, C6, D9, D10, D70, D73, D74, D75, D80	
D25	D33	B35, C1, D26, D27, G52	
D26	D33	B35, C1, D25, G52	D27
D27	D33	B35, C1, D25, G52	D26
D33	A12, B7, B11, B21, B35, C5, D10, D25, D26, D27	B16, D13	
D37	D48	A31, D8, D20, D69, F41	
D48	A31, B50, D8, D20, D37, D69, F41		
D65	A18, B14, B34, D17		
D69	D48, E4	A31, D8, D20, D37, F41	
D70	C2, C6, D9	D10, D24, D73, D74, D75, D80	
D73		C2, C6, D9, D10, D24, D70, D74, D75, D80	
D74	D9, D13	C2, C6, D10, D24, D70, D73, D75, D80	
D75	D13	C2, C6, D9, D10, D24, D70, D73, D74, D80	
D80		C2, C6, D9, D10, D24, D70, D73, D74, D75	
E4	A31, D20, D69, D74, F41		B3
F41	B3, D48, E4	D8, D20, D37, D69	A31
G52	C1	B35, D25, D26, D27	

## Data Availability

The datasets used and/or analyzed during the current study are available from the corresponding author on reasonable request.

## Supplementary Materials

The following supporting information can be downloaded at https://www.mdpi.com/article/10.3390/v16111747/s1, Figure S1: Heatmap of binding antibody levels over the observation period from 2018 to 2022; Figure S2: Bubble plot visualization of binding antibody levels over the observation period from 2018 to 2022, order of donors specific for each HAdV type; Figure S3: Correlation analysis of binding antibody levels of all donors against the different HAdV types.
