# Supplementary material for: Longitudinal Analysis of Binding Antibody Levels Against 39 Human Adenovirus Types in Sera from 60 Regular Blood Donors from Greifswald, Germany, over 5 Years from 2018 to 2022
Source: Viruses. 2024 Nov 7;16(11):1747. doi: 10.3390/v16111747 (PMC11598854; doi:10.3390/v16111747)
Supplement: Supplementary file 1 [file viruses-16-01747-s001.zip › viruses-3247922-supplementary.pdf]

Supplementary Figure S1

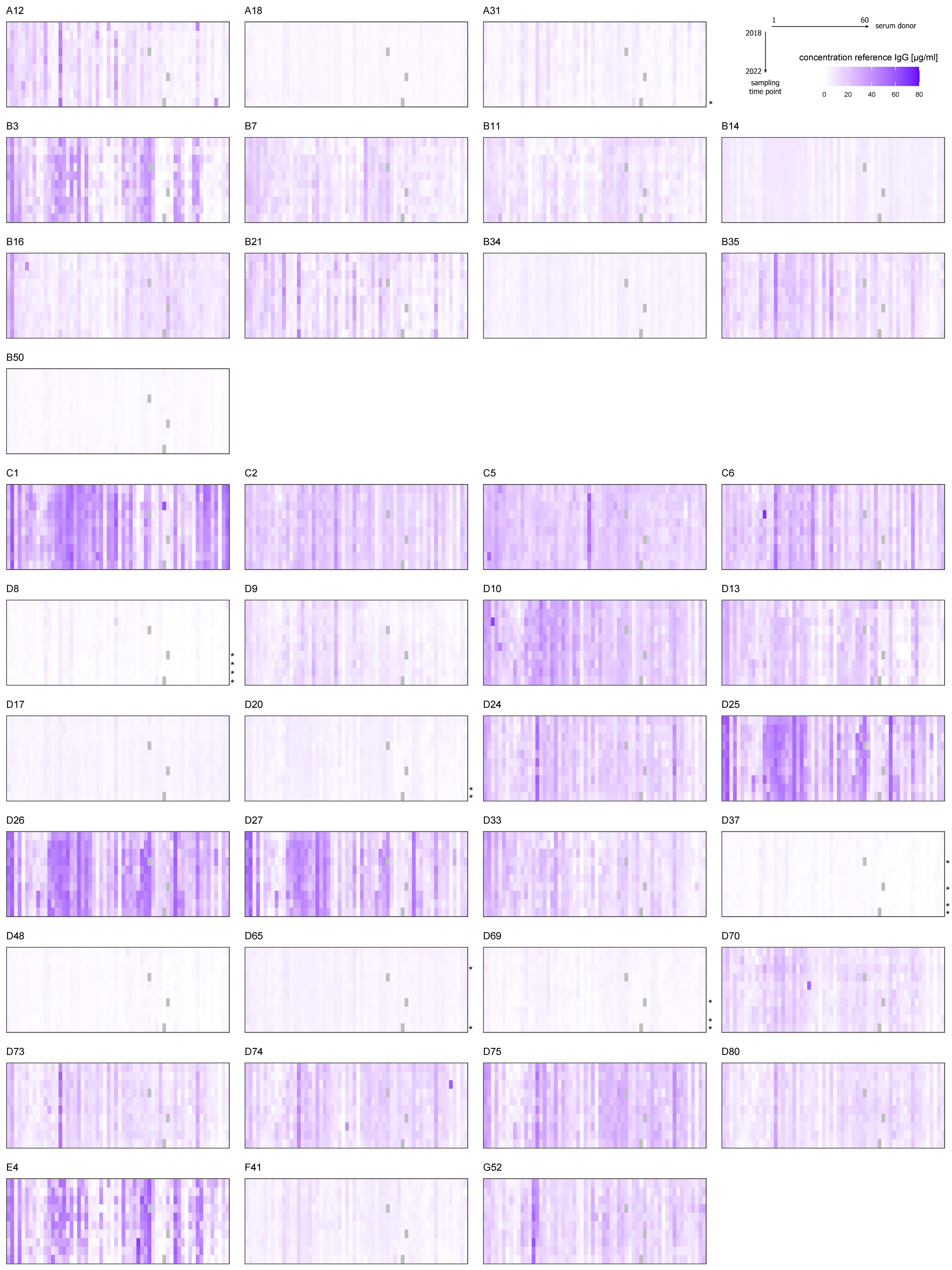

Heatmap of binding antibody levels over the observation period from 2018 to 2022. (legend continued on next page)

Heatmap of binding antibody levels over the observation period from 2018 to 2022. (continued legend) Binding antibody levels in sera were determined by ELISA and the binding antibody levels are represented by color in the heatmaps for the indicated HAdV types that show donors ordered by their arbitrary donor number arranged from left to right and the collection dates from 2018 to 2022 arranged from top to bottom. bAb: binding antibody. * indicates statistically significant differences of the antibody levels in the serum samples from the indicated year compared to antibody levels in the first serum samples from 2018 ($p < 0.05$, one-way analysis of variance on ranks with Dunn's multiple comparison test); no statistically significant differences were found for the comparison of the first set of samples from 2020 with samples from subsequent time points ($p > 0.05$).

Supplementary Figure S2

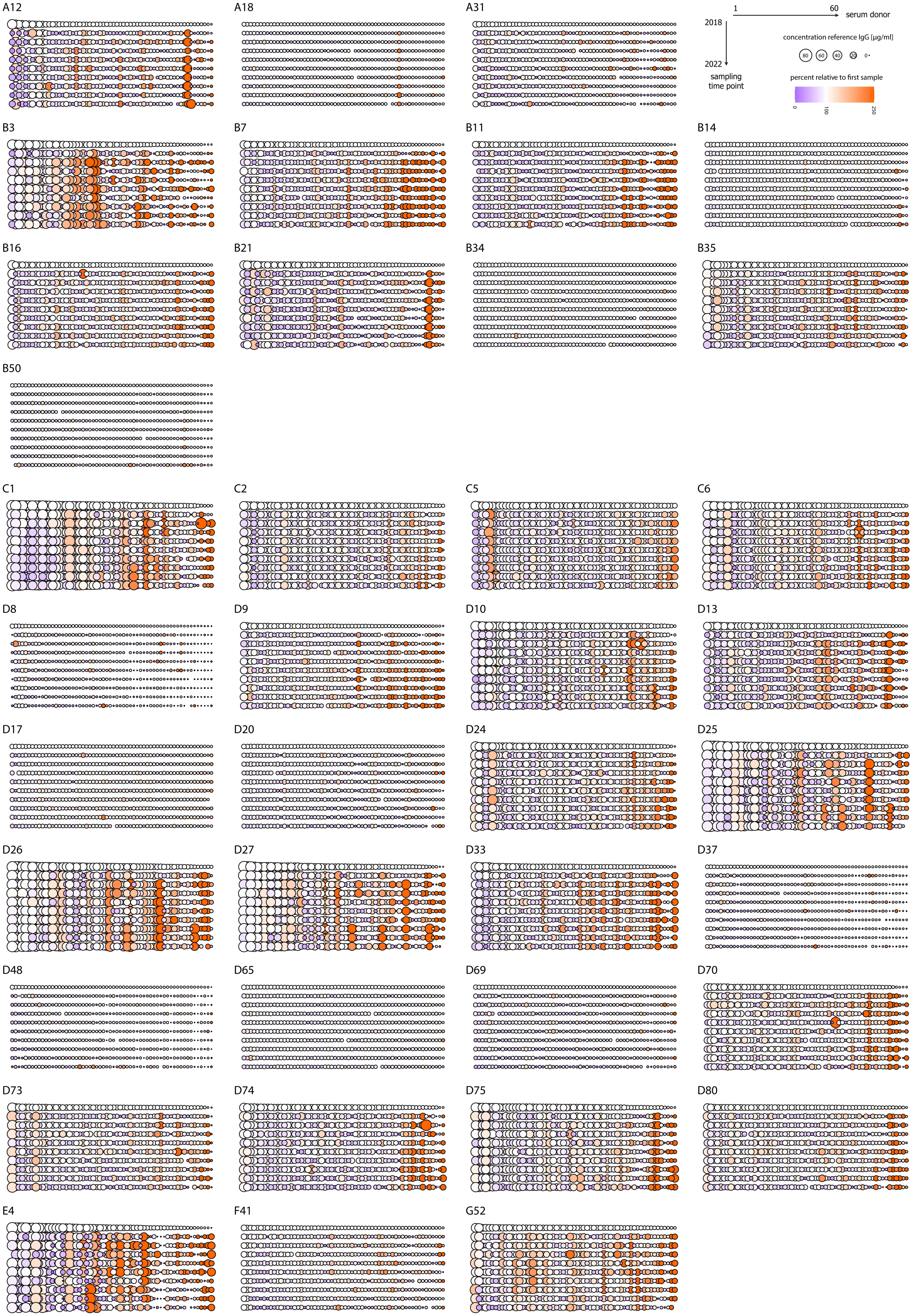

Bubble plot visualization of binding antibody levels over the observation period from 2018 to 2022, order of donors specific for each HAdV type. (legend continued on next page)

Bubble plot visualization of binding antibody levels over the observation period from 2018 to 2022, order of donors specific for each HAdV type. (continued legend)

Binding antibody levels against the indicated HAdV types as shown in Figure 2; the binding antibody levels are indicated by the size of the bubbles, the color indicates the relative change in percent compared to the respective level in the donor's first sample obtained in early 2018. The bubble plots show donors ordered according to the binding antibody levels in the first sample from 2018 against the indicated HAdV type arranged from left to right and the collection dates from 2018 to 2022 arranged from top to bottom. The order of the donors is different in each individual bubble plot.

Supplementary Figure S3

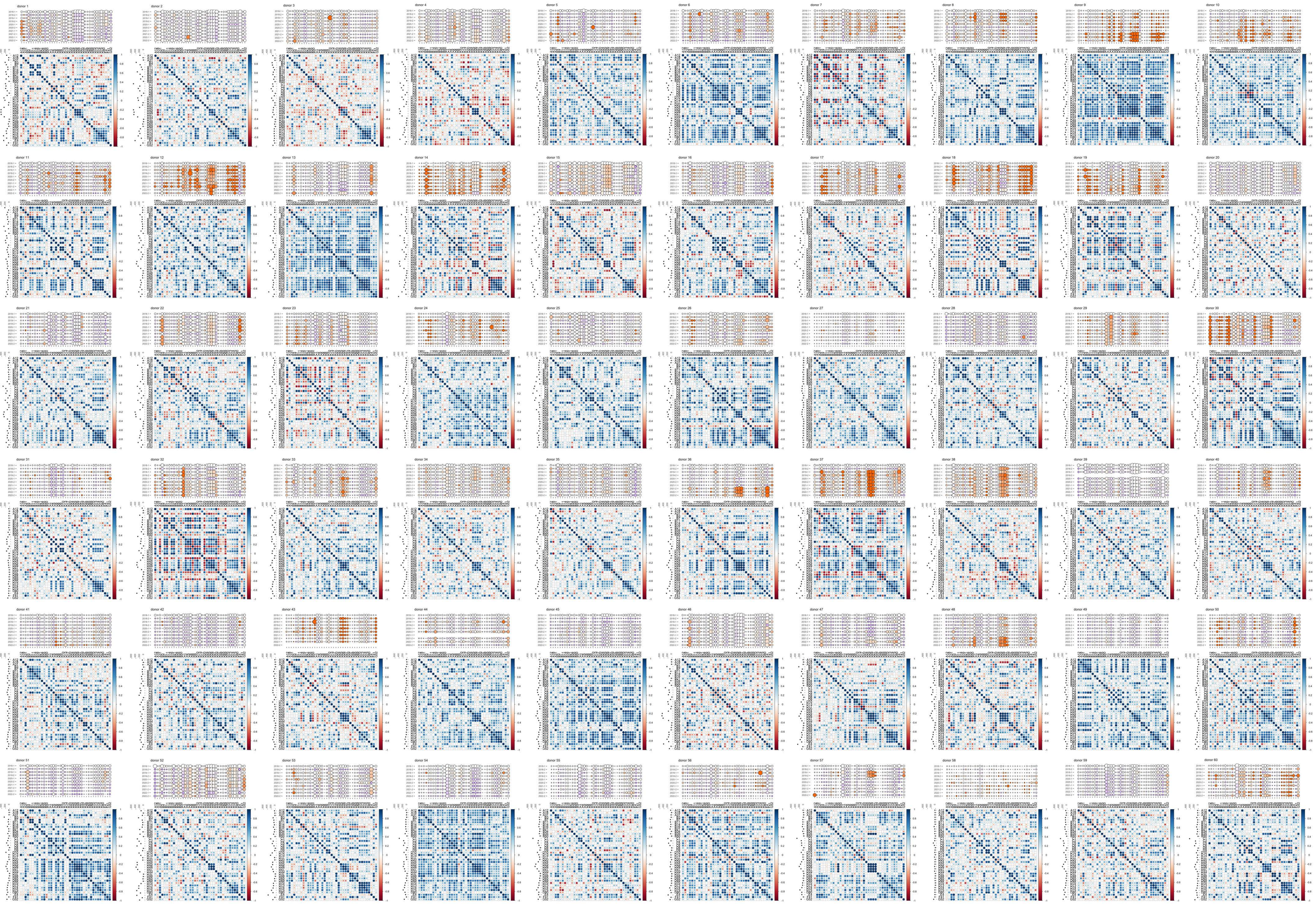

Correlation analysis of binding antibody levels of all donors against the different HAdV types. (legend continued on next page)

Correlation analysis of binding antibody levels of all donors against the different HAdV types. (continued legend)

A Pearson correlation analysis was performed for the binding antibody levels against the different HAdV types as shown in Figure 2 for each individual donor. Shown are the correlation maps for the indicated donors with the binding antibody levels shown as bubble plots as in Figure 2 on top of the correlation map and the area under the curve of the binding antibody levels against the respective HAdV type over the 5-year period shown on the left side of the correlation map.
